# Structural Analysis of a Non-Starch Polysaccharide Derived from Red Rice Bran and Its Immunomodulatory Properties

**DOI:** 10.3390/foods15142560

**Published:** 2026-07-21

**Authors:** Juan Liu, Dagang Chen, Xinqiao Zhou, Yangchao Ou, Ke Chen, Chanjuan Ye, Jie Guo, Chuanguang Liu

**Affiliations:** 1 Rice Research Institute, Guangdong Academy of Agricultural Sciences, Key Laboratory of Genetics and Breeding of High Quality Rice in Southern China (Co-Construction by Ministry and Province), Ministry of Agriculture and Rural Affairs/Guangdong Key Laboratory of Rice Science and Technology, Guangzhou 510640, China or ljane0505@126.com (J.L.); chendg@gdaas.cn (D.C.); 13428860986@139.com (X.Z.); chenke@gdaas.cn (K.C.); chanjuanye@163.com (C.Y.); 2 School of Food Science and Engineering, South China University of Technology, Guangzhou 510640, China; 202421027107@mail.scut.edu.cn

**Keywords:** red rice bran, polysaccharide, immunomodulatory, macrophage activation, proinflammatory cytokine

## Abstract

A polysaccharide with water solubility named RRBP was extracted from red rice bran (*Oryza sativa* L.), and its structural characterization was analyzed. The polysaccharide exhibited a monodisperse molecular weight distribution with an average of 51.4 kDa, and also comprised glucose (98.1%, *w*/*w*) and small amounts of arabinose, galactose, xylose, and mannose. Structural investigations using methylation analysis combined with UV and FT-IR spectroscopy demonstrated that RRBP is a structurally complex hyperbranched glucan characterized by an extensive branch with “→4)-α-d-Glcp-(1“→linkages constituting 30.6% of the backbone. RRBP demonstrated concentration-dependent stimulation of nitric oxide (NO) generation along with elevated secretion levels of IL-6 and TNF-α but showed minimal effect on IL-12 production in assays with RAW264.7 macrophage cells. Gene expression profiling verified the enhanced transcription of genes encoding iNOS and pro-inflammatory cytokines including IL-6, TNF-α, and IL-12 following RRBP treatment. Moreover, it is important to note that RRBP was able to promote M1 macrophage activation and exhibited no cytotoxicity, and this implied that RRBP could serve as a biocompatible pro-inflammatory immunostimulant. In summary, these findings suggested that RRBP is a distinctive glucan that possesses selective immunomodulatory properties and could be applied in the development of functional food products designed for targeted immune system modulation.

## 1. Introduction

As the primary by-product of rice processing, rice bran currently serves chiefly as a source material for producing rice bran oil and phytic acid derivatives in China, but its utilization efficiency is only 15% of total production. Most rice bran (approximately 85%) is used to make low-grade animal feed; thus, rice bran resources are being wasted to a large extent. Remarkably, this low-efficiency usage of rice bran indicates enormous potential to create new value-added products from agricultural by-products. Interestingly, the polysaccharides isolated and purified from rice bran have various biological activities and applications, but they have received less attention.

Rice bran polysaccharides (RBPs) extracted predominantly from defatted rice bran are complex heteropolysaccharides containing arabinose, galactose, glucose, mannose, rhamnose, and xylose in different ratios, and the α-1,6-glycosidic bond has been reported as a prominent structural theme [1,2]. In addition, the existence of α-1,4-glycosidic bonds has been confirmed using FT-IR and ^13^C NMR spectroscopic analysis [1]. Recent investigations have provided evidence that approaches for the sulfation of RBPs involving chlorosulfonic acid-pyridine have been applied extensively to append sulfate groups mainly on the hydroxyl positions of the C-6 and enhance the aqueous solubility characteristics and biological activities of RBPs. Moreover, the best parameters to strengthen the molecular charge distribution and spatial configurations of sulfated isoforms are a sulfation level between 0.81 and 1.29 and carbohydrate content ranging from 41.41% to 78% [3]. Bioprocessing using Grifola frondosa mycelium significantly enhances the therapeutic potential of RBPs by reducing their molecular weight (from 10^3^–10^4^ Da to 10^2^–10^3^ Da) and adjusting the proportions of the saccharide components [4]. These recent studies have revealed the use of effective strategies to design RBPs precisely according to the expected biomedical applications.

RBPs exhibit diverse biological abilities including antimicrobial, antioxidant, anticancer, and immune-regulatory properties. It has been shown that sulfated sRBP52 (DS = 1.29) displays appreciable antitumor activity by inhibiting Hep-G2 hepatoma and B16 melanoma cell proliferation through immune activation and direct cytotoxicity [3]. Other studies have shown that RBPs also possess antioxidant capacity through enhancing the activity of catalase (CAT) and superoxide dismutase (SOD), coupled with decreasing MDA accumulation [1]. Moreover, RBPs can also serve as metabolic regulators that ameliorate hyperlipidemia induced through the diet by downregulating lipid synthesis gene expression (SREBP-1C, FASN) and enhancing fatty acid oxidation markers (PPAR-α, CD36) in high-fat diet models. Remarkably, sulfated RBPs have a good effect on immunomodulation via macrophage activation and cytokine regulation (e.g., TNF-α, IL-6) [5]. Furthermore, RBPs also attenuate heavy metal toxicity by reducing cadmium accumulation and restoring gut microbiota balance [6]. All these findings reveal that RBPs play a crucial role in therapeutic interventions or nutraceutical applications.

Red rice is a variety of colored rice with the largest production in China. The content of vitamins and flavones in red rice is 14.23 times and 5.58 times that in ordinary brown rice. Although several rice bran polysaccharides (RBPs) have been reported with immunomodulatory activities, most of these studies focused on heteropolysaccharides composed of arabinose, galactose, xylose, and mannose in varying ratios, or on β-glucans with (1→3)- or (1→6)-linked backbones. To date, it has not yet been reported that any red rice bran-derived glucan is characterized by a →4)-α-D-Glcp-(1→ dominant backbone (30.6%) combined with such extensive branching complexity (11 distinct glycosidic linkage types) and a molecular weight of 51.4 kDa. Moreover, the selective immunomodulatory profile—potent induction of NO, TNF-α, and IL-6 with only minimal IL-12 elicitation—has not been previously documented for rice bran polysaccharides. Therefore, RRBP represents a structurally distinctive glucan from red rice bran with a unique immunomodulatory signature that distinguishes it from previously characterized rice bran polysaccharides. The polysaccharides of red rice bran also possess various bioactivities; however, there are few reports on the immune-regulatory activities of natural bran polysaccharides of red rice. Hence, this investigation focuses on extracting and refining a novel structurally complex glucan polysaccharide compound (RRBP) from red rice bran, conducting comprehensive structural characterization encompassing molecular weight parameters, monosaccharide configuration, UV spectral patterns, Fourier transform infrared spectroscopy (FT-IR) signatures, and methylation profiling. Furthermore, macrophage-based experimental models were employed to assess RRBP’s immunoregulatory potential. The elucidated structural characteristics and the immunomodulatory activity of RRBP provide a good basis for using it as a potential nutritional additive with immunologically active properties.

## 2. Materials and Methods

### 2.1. Materials and Reagents

The material (*Oryza sativa indica* L. cv. Nanhong No.8) was collected from agricultural research plots in Huizhou City, Guangdong Province during the 2023 growing season, exhibiting a moisture content of 13%. The red rice bran was milled using a rice milling machine (Yamamoto, JP), and every 30 g of red rice was milled for 30 s, so that the surface of the milled red rice was free from red rice bran, and the red rice bran powder was devoid of white endosperm powder. Certified monosaccharide reference materials (≥99% purity), Escherichia coli-derived lipopolysaccharide (O111:B4 strain), and the cell viability indicator MTT (3-(4,5-dimethylthiazol-2-yl)-2,5-diphenyltetrazolium bromide) were acquired from Sigma-Aldrich (St. Louis, MO, USA). Cellular analysis reagents including the CCK-8 proliferation assay kit and nitric oxide detection system were commercially sourced from Beyotime Biotechnology (Shanghai, China). Murine cytokine quantification employed ELISA kits from Neobioscience (Shenzhen, China).

### 2.2. Extraction and Purification of RRBP

Red rice bran was powdered through mechanical grinding and subjected to delipidation process via absolute ethanol treatment under continuous agitation for 2 h. After centrifugation (6000× *g*, 10 min), the residue underwent hot water extraction (1:10, *w*/*v*) at 70 °C for 4 h. The extract (clear solution) was evaporated under vacuum and precipitated with ethanol (60%, 4C, 12 h). The polysaccharide-containing precipitate was centrifuged at 7000× *g* for 15 min, then dried to yield crude polysaccharide fractions.

After dissolving in deionized water, the crude polysaccharides were hydrolyzed with papain for 12 h. The aqueous extract was sequentially processed by adding chloroform and butanol (4:1), mixing and centrifuging to obtain the separated aqueous hydrophilic fraction. After that, the solution was mixed with an equal volume of petroleum ether, and the higher-density aqueous phase was successfully decanted off. The obtained solution was subjected to further refinement using AB-8 Macroporous Adsorption resin by shaking the mixture intensely for better adsorption, followed by shaking overnight (12 h). The filtered solution was dialyzed using a 3 kDa dialysis membrane for 2 days to remove unwanted low-molecular-weight materials, and after lyophilization, the final polysaccharide compound was obtained.

The initial polysaccharide samples were dispersed in deionized water and subjected to centrifugation at 10,000× *g* for 10 min. The resulting supernatant underwent purification through an AKTA purification system (GE, explorer 100) utilizing a DEAE Sepharose Fast Flow column (26 mm × 400 mm) (Sigma-Aldrich, St. Louis, MO, USA) with a flow rate maintained at 4 mL min^−1^. Sequential elution was performed using deionized water followed by stepwise NaCl concentrations (0.1, 0.2, and 0.3 M). Fractions collected between 7 and 19 min underwent concentration and dialysis through a 3000 Da molecular weight cutoff membrane for 72 h to eliminate ionic contaminants. Following ion-exchange processing, the polysaccharide fractions were rehydrated in deionized water and centrifuged at 10,000× *g* for 10 min. The clarified supernatant was subsequently fractionated using the AKTA purification system (GE, explorer 100) equipped with a Sephacryl S-400HR column (26 mm × 1000 mm) operating at a 1 mL min^−1^ flow rate. Elution involved collection after passing 1.5 column volumes of deionized water. The final concentrated and lyophilized fractions were prepared for subsequent analytical procedures.

### 2.3. Molecular Weight Measurement

Molecular weight was measured using a combination of gel-permeation chromatography with a Thermo UltiMate 3000 quaternary pump system. Separation was achieved with the following columns in series; two T-shaped Shodex Ohpak SB-805 HQ and SB-803 HQ columns (300 × 8 mm, 6 µm particles; Resonac Corporation, Tokyo, Japan) at 35 °C. The data were calculated using the ASTRA 6.1 software.

### 2.4. Ultraviolet Spectrum Scan and Infrared Spectrum Analysis

RRBP samples were subjected to UV spectral scanning in the 200–1000 nm range by microplate scanning of quartz microplates at 1 nm intervals using a Thermo Multiskan GO microplate reader (Thermo Fisher Scientific, Waltham, MA, USA), with solvent blank background corrections. Infrared characterization was carried out on a Nicolet iS50 spectrometer fitted with DTGS detector (Thermo Fisher Scientific, Waltham, MA, USA) and 5 mg lyophilized polysaccharide was milled with 200 mg KBr and pressed into pellets under vacuum (10 t cm^−2^, 1.2 s). The infrared spectrum was recorded with resolution of 450–4000 cm^−1^ at 4 cm^−1^ resolution with 64 scan accumulations.

### 2.5. Monosaccharide Composition Determination

Carbohydrate monomer analysis was conducted using an ICS 5000+ ion chromatography system (Thermo Fisher Scientific, Waltham, MA, USA) equipped with electrochemical detection, following acid hydrolysis and derivatization procedures.

The chromatographic separation utilized a Dionex™ CarboPac™ PA20 analytical column (3.0 × 150 mm, 10 μm particle size). Samples were introduced via a 5 μL injection volume. Mobile phase compositions included (A) deionized water, (B) 0.1 M sodium hydroxide and (C) 0.1 M sodium hydroxide with 0.2 M sodium acetate, delivered at a constant flow rate of 0.5 mL min^−1^ at 30 °C. The elution program was as follows: 95% A, 5% B, 0% C initial conditions for 26 min (isocratic), then an isocratic regime of 85% A, 5% B, 10% C from 26 to 42 min, a step to 60% A and 40% C at 42.1 min, a step to 60% A and 40% B at 52 min and return to initial conditions (95% A, 5% B) for 8 min (column re-equilibration) between 52.1 and 60 min.

### 2.6. Methylation Analysis

Purified RRBP was completely dissolved in DMSO. Hydrolysis of the permethylated derivatives was carried out in 2 M TFA at 121 °C for 90 min and further reduced with sodium borodeuteride (NaBD_4_). Subsequently, acetic anhydride acetylation was performed at 100 °C for 150 min, and the resulting alditol acetates were dissolved in chloroform for the GC-MS determination on the Agilent 6890A-5977B setup using an Agilent BPX70 capillary column (30 m × 0.25 mm × 0.25 μm, SGE Analytical Science, Ringwood, VIC, Australia). Ultra-high-purity helium (carrier gas, 10:1 split ratio) was applied at a 1 μL injection volume, starting from 140 °C (2 min hold), then 10 °C min^−1^ ramp to 230 °C (3 min hold). Standard analytical conditions applied to mass spectral detection. Full-scan MS acquisition spanned m/z 50–350 in SCAN mode.

### 2.7. Cell Culture and Cytotoxicity Determination

RAW264.7 macrophage cell line, provided by Zhujiang Hospital of Southern Medical University with an initial cell concentration of 10^5^–10^6^ cells mL^−1^, were cultured firstly in 90%DMEM with 9% FBS medium with 1% penicillin-streptomycin antibiotic mixture After 12 h of incubation, the cultures were grown at 37 °C in a humidified (95%) atmosphere with (5%) CO_2_ to obtain a consistent monolayer and metabolic balance. Fresh DMEM with purified RRBP polysaccharides (0, 0.1, 0.2, 0.4, 0.8, 1.5 or 2.0 mg mL^−1^) was then replaced as the culture medium and the cells were incubated for 24 h. Following treatment, cell viability analysis utilized a two-fluorescence staining technique wherein viable cells were labeled by the membrane permeant fluorophore Calcein-AM (10 µg mL^−1^) exhibiting green fluorescence due to intracellular esterase action.

In order to discriminate between viable and non-viable cells, we first added the SYTOX Green nucleic acid stain (1 μM, Thermo Fisher Scientific) which stains the membrane of cells with permeability defects; propidium iodide, a red-fluorescent DNA dye, was applied; both cell groups’ nuclei were then stained with blue-fluorescent DNA targeting Hoechst 33342 simultaneously. Three channels of fluorescence imaging were obtained by an inverted microscope system equipped with specific optical filters for the different staining patterns.

All experiments were performed with three independent biological replicates, each containing three technical replicates. All data were displayed as the mean ± standard error (SE). Statistical analyses were performed using SPSS version 26 (SPSS, Inc., Chicago, IL, USA) via one-way analysis of variance (ANOVA) and Duncan’s test was used to test the significance of the difference (*p* < 0.05).

### 2.8. Cell Proliferation Assay

The proliferative activity of RRBP- treated RAW264.7 cells was analyzed spectrophotometrically with a CCK-8 assay kit. Cells were exposed to RRBP for 24 h for cytotoxicity experiments. Cells were thoroughly removed from the culture supernatant and phenol red-free DMEM supplemented with 10% (*v*/*v*) solution of CCK-8 was added. Cellular dehydrogenase activity from the viable cells during an hour of incubation under normal culture conditions converted the tetrazolium salt into water-soluble formazan. A microplate reader measured the absorbance at 570 nm, and the optical density rose in direct proportion to viable growing cells after RRBP exposure.

### 2.9. Cell Differentiation Measurement

To determine the influence of RRBP on polarization, a protocol for fluorescence- based differentiation was performed. RAW264.7 cells treated with RRBP for 24 h were sequentially subjected to fixation with 4% paraformaldehyde (30 min), immersion in 0.2% TritonX-100 in PBS (10 min), and blocking with 5% goat serum (1.5 h) to avoid nonspecific antibody binding. Dual-immunostaining was performed with primary antibodies at identical dilutions (1:250) overnight. M1 polarization was identified using rabbit anti-iNOS, and M2 polarization with mouse anti-CD206. Following extensive washes in PBS, fluorescence was performed with APExBIO’s HyperFluor 488-conjugated anti-rabbit IgG (1:500) and Bioss’s Cy5-labeled anti-mouse IgG (bs-0296G-Cy5, 1:500) (1 h incubation at RT in darkness). After 15 min of DAPI nuclear staining, imaging via fluorescence microscope distinguished M1 (green) from M2 (red) cells.

The counterstaining was with DAPI solution (300 nM) for another 15 min. Fluorescent pictures were taken by an inverted microscope: M1 macrophages appeared green under bright fluorescence, and M2 macrophages exhibited red fluorescence.

### 2.10. Determination of NO Level and Secretion of Pro-Inflammatory Cytokines

RAW264.7 murine macrophages were grown in DMEM with 10% heat-inactivated FBS plus 100 U mL^−1^ penicillin and 100 µg mL^−1^ streptomycin under controlled culture conditions (37 °C, 5% CO_2_). During experimental procedures, log-phase cells were plated at a density of 100 µL well^−1^ and cultured to reach confluent monolayers in 24 h. After removing medium, test solutions with RRBP at dilutions (125–1000 µg mL^−1^) or LPS control (50 µg mL^−1^) were added and subsequent analyses were conducted.

Experimental conditions were tested across four technical replicates. Supernatants (from cells treated for 24 h) were collected carefully, centrifuged to clarify the liquid (300× *g*, 5 min spin), aliquoted and stored. The nitric oxide was determined spectrophotometrically based on a diazotization of the Griess reaction (absorption at 540 nm). Secreted cytokine levels at the same time points were additionally quantified with species-specific commercial ELISA kits according to manufacturers’ instructions; special consideration was taken in the case of TNF-α.

To exclude potential endotoxin interference, endotoxin levels in RRBP were determined using a LAL chromogenic endpoint assay (Thermo Fisher). Additionally, RRBP or LPS (positive control) was pre-incubated with polymyxin B (10 μg mL^−1^, 37 °C, 30 min) prior to macrophage treatment to neutralize any trace LPS contamination.

All experiments were performed with three independent biological replicates, each containing three technical replicates. All data were displayed as the mean ± standard error (SE). Statistical analyses were performed using SPSS version 26 (SPSS, Inc., Chicago, IL, USA) via one-way analysis of variance (ANOVA) and Duncan’s test was used to test the significance of the difference (*p* < 0.05).

## 3. Results

### 3.1. Extraction and Purification of RRBP

The water-soluble polysaccharides from rice bran were obtained through heated aqueous extraction. Sequential purification steps involving anion-exchange chromatography followed by gel filtration chromatography were employed for RRBP refinement. The predominant fraction by mass recovery was isolated for subsequent analysis. Post-purification yields through ionic separation and molecular sieving reached 35.4% and 55.2% respectively.

### 3.2. Molecular Weight and Monosaccharide Composition of RRBP

**Figure 1:**
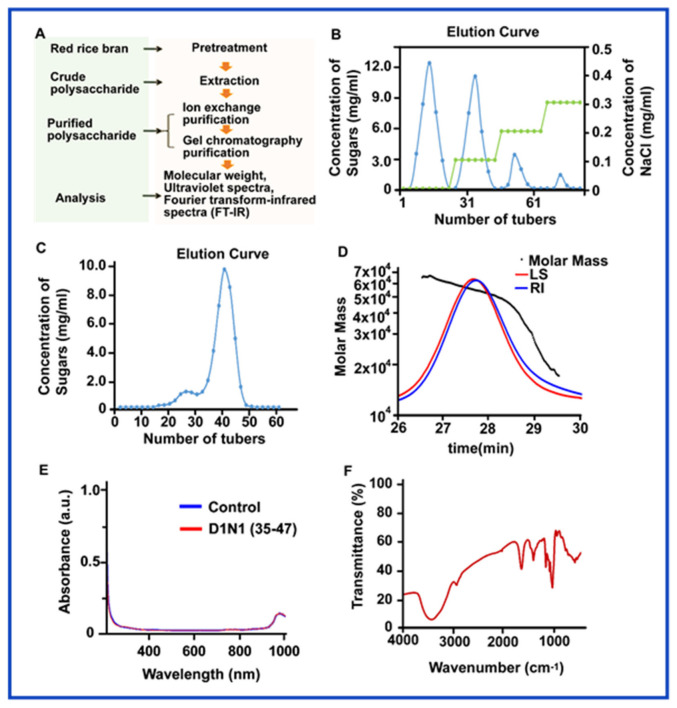
Purification and structural characterization of the RRBP. (**A**) The experimental procedure of the RRBP. (**B**) Chromatography of the polysaccharides by ion exchange purification. (**C**) Chromatography of the polysaccharides by gel chromatography purification. (**D**) The molecular weight analysis of RRBP. (**E**) The UV spectra of RRBP. (**F**) The FT-IR spectra of RRBP.

**Figure 2:**
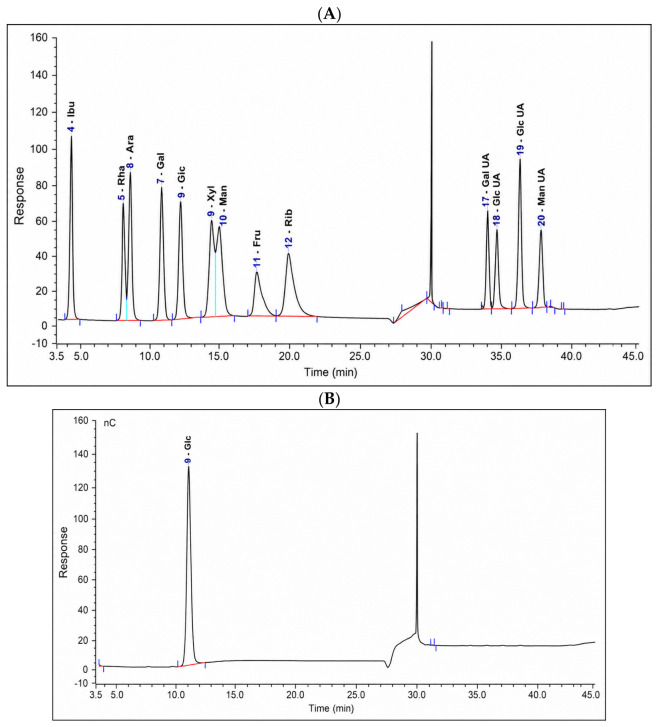
Gas chromatograms of standard monosaccharides (**A**) and RRBP (**B**).

Gel permeation chromatography analysis revealed RRBP’s homogeneous molecular mass of 51.35 kDa, evidenced by a unimodal elution profile (Figure 1D). Compositional analysis demonstrated RRBP comprised glucose (98.06%) with trace arabinose, galactose, xylose and mannose at molar ratios of 0.43:0.5:0.32:0.31, confirming its predominant glucan structure with glucose constituting over 98% (Figure 2).

### 3.3. Spectroscopic Characteristics

UV spectral analysis of RRBP (Figure 1E) demonstrated negligible absorbance across the 200–400 nm range, suggesting minimal presence of contaminating substances including pigments, nucleic acids, and proteins. The FT-IR spectral profile presented in Figure 1F revealed characteristic absorption bands. A prominent absorption band spanning 3600–3200 cm^−1^ corresponded to hydroxyl group stretching vibrations, a diagnostic feature of polysaccharide compounds. FT-IR spectrum displayed a typical sugar O–H band at 3434 cm^−1^, C–H stretching at 2929 cm^−1^, and C–O stretching at 1025 cm^−1^.

### 3.4. Methylation Determination of RRBP

**Figure 3:**
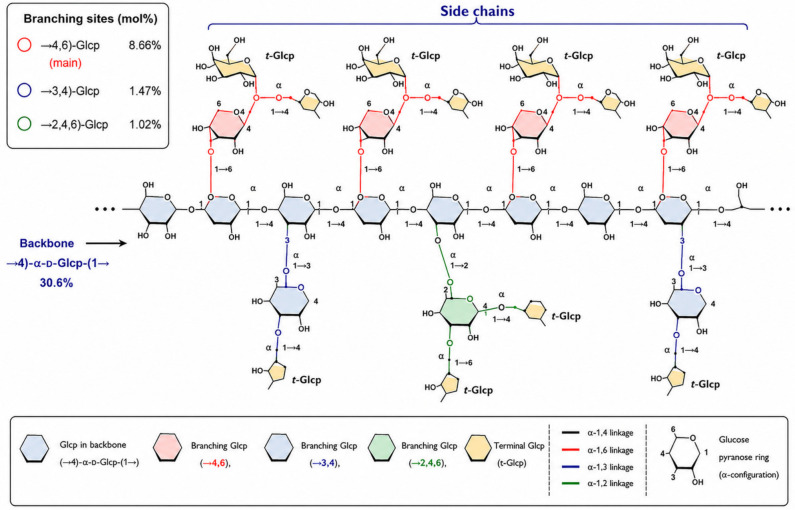
Schematic representation of the proposed structure of RRBP. The backbone consists of →4)-α-D-Glcp-(1→ linkages (highlighted in bold). Branching occurs primarily at O-6 positions (→4,6)-Glcp, with minor branches at O-3 and O-2 positions. Terminal glucose residues (t-Glcp) represent the non-reducing ends. The percentages indicate the molar ratios of each linkage type determined by methylation-GC-MS analysis (Table 1). Glcp: glucopyranose.

To elucidate the glycosidic linkage type in RRBP, the polysaccharide was subjected to methylation with CH_3_I as the methylating agent followed by GC–MS analysis. The results showed that eleven glycosidic bonding types were present in RRBP and the specific bonding information was listed systematically as shown in Table 1. Methylation analysis revealed t-Glcp, 3-, 2-, 6-, 4-linked Glcp, plus 3,4-, 2,4-, 3,6-, 4,6-, 2,6- and 2,4,6-branched Glcp residues. However, the 4-linked Glcp composition had the highest content (30.64% molar ratio), which suggested that this was likely the major structural backbone. The identification of multiply substituted units including 3,4-, 4,6- and 2,4,6-branched Glcp residues demonstrated that complex branching was also present in the polysaccharide structure. Based on the methylation analysis data presented in Table 1, the structural model of RRBP was constructed according to the following principles. The →4)-Glcp residue, exhibiting the highest molar ratio (30.64%) among all non-terminal linear linkages, was designated as the primary backbone constituent. Branching nodes were identified as residues substituted at multiple positions, including 3,4-, 4,6-, and 2,4,6-linked Glcp, with the →4,6)-Glcp residue (8.66%) representing the predominant branching motif, indicating that O-6 of the backbone glucose units serves as the primary attachment site for side chains. Terminal Glcp (t-Glcp, 33.36%) constitutes the non-reducing ends of the polysaccharide, while the remaining linear residues (3-, 2-, and 6-linked Glcp) form the extension segments of side chains. The coexistence of 11 distinct linkage types, combined with the high proportion of terminal residues and multiple branching nodes, collectively supports a hyperbranched architecture with a →4)-α-D-Glcp-(1→ backbone and diverse side-chain structures. A schematic representation of this proposed model is provided in Figure 3.

### 3.5. The Fluorescence Images of Resulting Cells Activity

Functioning as mononuclear phagocytes, macrophages serve as crucial components of the body’s first line of defense, participating in immune reactions, inflammatory processes, and numerous homeostatic mechanisms [7]. These carbohydrate-based compounds demonstrate immunoregulatory properties through their ability to modulate cytokine and chemokine secretion in macrophages [8]. The immunostimulatory potential of specific polysaccharides has undergone extensive investigation, with notable examples including Astragalus-derived polysaccharides, Dendrobium-based carbohydrate complexes, and Ganoderma lucidum polysaccharide extracts [9,10,11]. Nevertheless, the immunomodulatory potential of rice-bran polysaccharides remains largely unexplored. Given the established correlation between cellular viability and immune response regulation, initial evaluation focused on RRBP’s cytotoxic effects. RAW264.7 murine macrophages were employed as experimental models, maintained under standard culture conditions (37 °C, 5% CO_2_) for 12 h stabilization. Subsequently, cells were exposed to varying concentrations of RRBP during a 24 h incubation period. Cellular viability was determined through a standardized fluorescence-based viability assessment using triple-staining methodology: untreated culture medium served as the negative control, with viable cells labeled by Calcein-AM (green fluorescence), non-viable cells identified through propidium iodide (PI) uptake (red), while nuclear chromatin was counterstained with Hoechst 33342 (blue).

**Figure 4:**
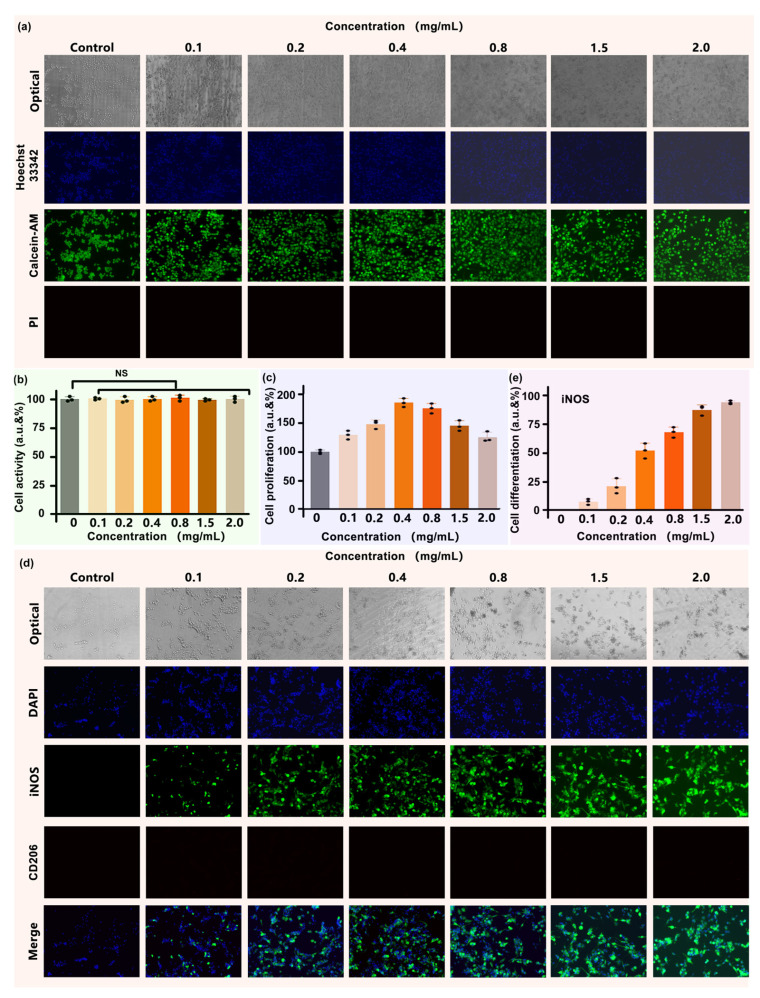
The fluorescence images of the resulting cell activity. (**a**) Optical and fluorescence images showed the viability profile of RAW264.7 cells (live and dead cells were stained with Calcein AM and propidium iodide, respectively) incubated with different concentrations of RRBP (0, 0.1, 0.2, 0.4, 0.8, 1.5, 2.0 mg mL^−1^). (**b**) Statistical analysis of RAW 264.7 cell viability. (**c**) Effect of different concentrations of RRBP on RAW264.7 cell proliferation. (**d**) Optical and fluorescence images and statistical analysis (**e**) showed the effects of different concentrations of RRBP on the differentiation of RAW264.7 cells (M1 were stained with iNOS (green), M2 were stained with CD206 (red), cell nucleus were stained with DAPI (blue)). Scale bar: 30 μm. Data are presented as mean ± SD from three independent biological replicates (*n* = 3). Statistical significance was determined by one-way ANOVA followed by Duncan’s test. *p* < 0.05 was considered statistically significant versus the control group.

The cellular fluorescence patterns illustrating metabolic activity are presented in Figure 4a. Following 24 h exposure to polysaccharide compounds, all experimental samples showed minimal red fluorescence signals, demonstrating RRBP’s non-cytotoxic properties. Quantitative assessments demonstrated cellular viability levels exceeding 98% across treatment groups, comparable to control specimens (Figure 4b). Notably, polysaccharide supplementation markedly enhanced green fluorescence intensity relative to the non-supplemented control group. The fluorescence response exhibited a biphasic concentration dependence, with initial enhancement followed by attenuation at higher polysaccharide concentrations, indicative of optimal proliferative stimulation within specific dosage ranges. For precise evaluation of RRBP’s proliferative effects, cellular density measurements were conducted following co-culture with serially diluted RRBP solutions.

Cell viability was assessed via CCK-8 assay. As illustrated in Figure 4c, peak proliferative activity occurred at polysaccharide concentrations near 0.4 mg mL^−1^, beyond which cellular growth showed progressive suppression with increasing dosage. This concentration-dependent inhibitory pattern contrasts with previous findings demonstrating linear growth enhancement effects observed with other biologically active polysaccharides.

Macrophage activation serves as a crucial defense mechanism against foreign pathogens and particulate matter [12]. When exposed to external agents, macrophages initiate chemotactic migration and enhance phagocytic functions, then polarize into pro-inflammatory M1 or anti-inflammatory M2 phenotypes to eliminate invaders through innate immunity pathways. However, excessive differentiation of macrophages will seriously affect cell proliferation. Therefore, we hypothesized that the inhibition of proliferation by high concentrations of RRBP is caused by the activation and differentiation of macrophages. To verify this hypothesis, after co-incubation of gradient concentrations of RRBP with cells, standard differentiation staining agents were used for calibration. M1 was labeled with green fluorescent iNOS, M2 was labeled with red fluorescent CD206, and nuclei were DAPI-stained. Figure 4d shows typical fluorescence images of cell differentiation. With increasing dose of RRBP, the proportion of green fluorescent cells gradually increased, and no red fluorescent cells were observed in all samples. This finding indicates that RRBP promoted the differentiation of RAW264.7 cells into the M1 phenotype. Statistical results showed that 90% of cells differentiated into M1 macrophages at 2.0 mg mL^−1^ RRBP, while at the optimal proliferation concentration (~0.4 mg mL^−1^), at least 50% of cells had differentiated into M1 macrophages (Figure 4e). Although RRBP lacks cytotoxicity and does not induce cell death, high concentrations of RRBP significantly promote macrophage differentiation into the M1 phenotype, thereby inhibiting cell proliferation. Therefore, as a potential functional food with immunomodulatory effects, RRBP at an appropriate concentration positively influences cell proliferation and differentiation, suggesting potential applications in immunomodulatory contexts that warrant further in vivo investigation.

### 3.6. Effects of RRBP on NO and Key Pro-Inflammatory Cytokines

The novelty of the present study lies in three distinctive aspects: the use of red rice bran as an underexplored polysaccharide source, the structurally complex hyperbranched glucan architecture (→4)-α-D-Glcp-(1→ backbone, 11 linkage types, 16.63% branching density, Mw 51.4 kDa), and a selective immunomodulatory profile (potent TNF-α/IL-6/NO with minimal IL-12) coupled with M1 polarization without cytotoxicity. To substantiate this novelty, we compared RRBP with representative immunomodulatory polysaccharides. MGN-3/Biobran is an arabinoxylan heteropolysaccharide that enhances phagocytosis and induces TNF-α/IL-6/NO [13], whereas RRBP is a pure glucan with a distinct cytokine signature. RON/SPR-901 is a nearly linear α-1,6-glucan with only ~5% branching [14], contrasting sharply with RRBP’s highly branched topology. RBP0 possesses a →4)-α-Glcp-(1→ backbone similar to RRBP but has a much lower molecular weight (4.57 × 10^3^ Da) and exhibits antioxidant rather than immunostimulatory activity [15]. Rice bran feruloylated oligosaccharides induce cytokines but are oligosaccharides, not high-Mw polysaccharides [16]. Cereal β-glucans (e.g., barley β-(1,3)(1,4)-glucans) activate macrophages via Dectin-1 but are β-configured [17], fundamentally different from RRBP’s α-glucan structure. Fungal glucans, such as lentinan, exhibit β-(1,3)-backbones with β-(1,6)-branches and typically elicit robust IL-12 responses via T-cell activation [18,19], whereas RRBP induces only minimal IL-12. Collectively, this comparative analysis confirms that RRBP’s structural and functional combination—hyperbranched α-glucan with a →4)-backbone, high branching density, and selective IL-12-sparing immunostimulation—has not been reported for any rice-derived, cereal-derived, or fungal polysaccharide to date.

The structure–activity relationship of RRBP warrants brief consideration. Its →4)-α-D-Glcp-(1→ backbone (30.6%) differs markedly from the immunostimulatory β-(1→3)-glucans reported for fungi and cereals, which are well-established Dectin-1 agonists [18]. Although α-glucans are less studied in this context, the high branching density (16.63%) and the presence of multiple branching nodes may facilitate multivalent interactions with macrophage surface receptors, such as TLR2, TLR4, or scavenger receptors, potentially promoting receptor clustering and downstream signaling [13,16]. The moderate molecular weight (51.4 kDa) may also be relevant; polysaccharides below 10 kDa often exhibit diminished receptor cross-linking, while those exceeding 100 kDa may suffer from steric hindrance or poor solubility, both of which could compromise immune recognition. Moreover, the selective IL-12-sparing effect observed with RRBP may reflect its unique branching architecture, which could engage signaling pathways that favor NF-κB activation while limiting IRF-dependent IL-12 transcription. Although these hypotheses require direct experimental validation, they provide a structural framework for understanding RRBP’s distinctive immunomodulatory profile.

Given RRBP’s pro-inflammatory immunostimulatory activity, its potential roles in conditions associated with macrophage polarization, such as tissue repair and tumor immunity, merit future exploration. Experimental evidence demonstrated RRBP’s regulatory influence on nitric oxide concentrations and inflammatory mediators that appeared crucial in the control of homeostatic response processes associated with the regeneration of damaged tissue and neoplastic progression.

**Figure 5:**
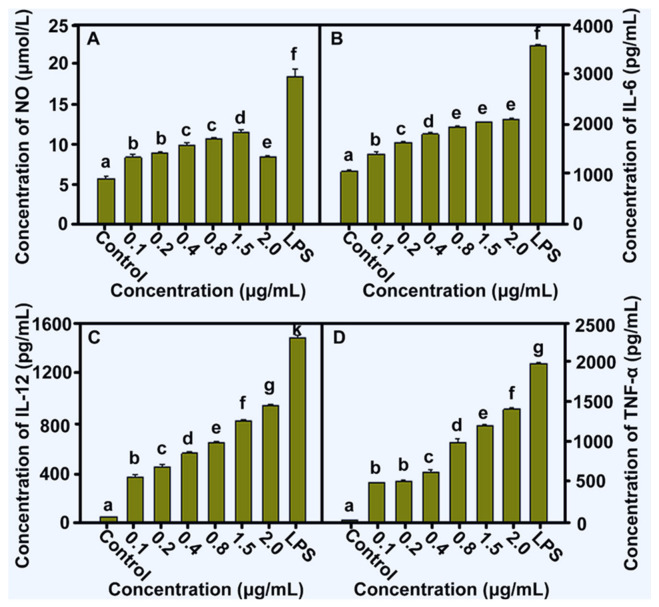
Effects of RRBP on the levels of NO and pro-inflammatory cytokines. (**A**–**D**) refer to NO, IL-6, IL-12 and TNF-α, respectively. Values with no letters in common are significantly different (*p* < 0.05). Data are presented as mean ± SD from three independent biological replicates (*n* = 3). Statistical significance was determined by one-way ANOVA followed by Duncan’s test. *p* < 0.05 was considered statistically significant versus the control group.

**Figure 6:**
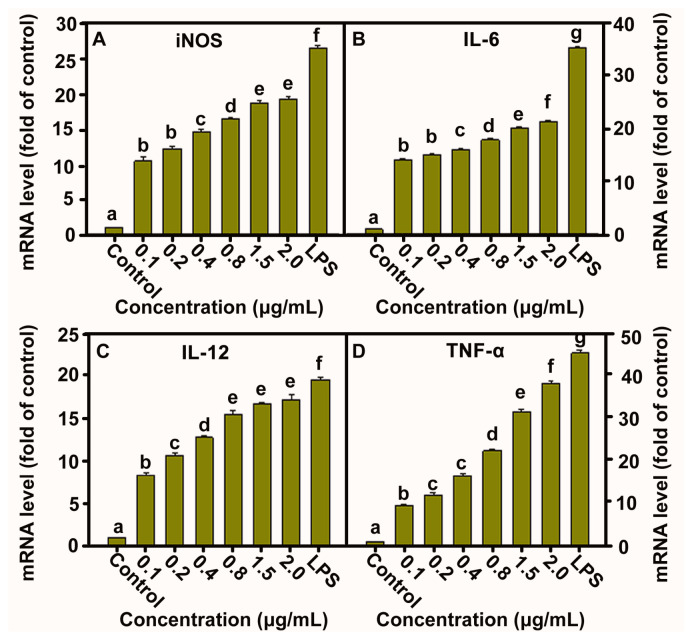
Effects of RRBP on the mRNA expression of genes related to inflammation response. (**A**–**D**) refer to iNOS, IL-6, IL-12 and TNF-α, respectively. Values with no letters in common are significantly different (*p* < 0.05). Data are presented as mean ± SD from three independent biological replicates (*n* = 3). Statistical significance was determined by one-way ANOVA followed by Duncan’s test. *p* < 0.05 was considered statistically significant versus the control group.

The LAL assay confirmed that the endotoxin levels in the final RRBP preparation were <0.05 EU/mL. Polymyxin B neutralization did not diminish RRBP-induced NO or cytokine production (*p* > 0.05), while completely abrogating LPS-induced responses (*p* < 0.01), confirming that the observed immunostimulatory effects are intrinsic to RRBP rather than artifacts of LPS contamination. The experimental data presented in Figure 5A–D demonstrate RRBP’s regulatory effects on macrophage-derived nitric oxide and inflammatory mediators. Figure 5A reveals a concentration-dependent elevation in NO synthesis following RRBP exposure, with maximal production observed at 500 μg mL^−1^ (*p* < 0.05 versus control). Figure 5B illustrates progressive enhancement of IL-6 secretion across treatment groups, where the 1000 μg mL^−1^ dosage group exhibited substantially higher cytokine levels compared to lower concentrations. As shown in Figure 5C, RRBP induced a moderate but significant concentration-dependent increase in IL-12, albeit at substantially lower absolute levels compared to TNF-α and IL-6, and significant differences were observed only at higher concentrations, suggesting that RRBP is selective in its regulation of different cytokines. Figure 5D documents a direct correlation between RRBP concentration and TNF-α secretion levels. This muted IL-12 response, contrasted with robust TNF-α/IL-6 production, suggests that RRBP does not overwhelmingly drive Th1-polarizing responses. As demonstrated in Figure 6, RRBP exerted gene expression modulation on inflammatory mediators at the transcript level. RRBP significantly upregulated the mRNA transcription of nitric oxide synthase (iNOS) (*p* < 0.05), which is consistent with the increase in NO secretion level, suggesting that RRBP promoted NO synthesis by enhancing the transcription of iNOS (Figure 6A). Transcriptional activation was also observed for pro-inflammatory cytokine genes including IL-6, IL-12 and TNF-α (*p* < 0.05), confirming RRBP’s direct trans-regulatory capacity for inflammatory mediators at the gene expression level.

In the current work, the authors have shown how rice bran polysaccharides (RRBP) possess strong immunomodulatory activity on murine macrophages, predominantly via concentration-dependent induction of proinflammatory cytokines and control of gene expression at the signaling network level. As shown in Figure 5A–D, RRBP evoked NO, IL-6 and TNF-α release in a dose-ascending fashion with a relatively weak induction of IL-12. This may indicate that RRBP selectively triggered the immune pathways (e.g., NF-κB for TNF-α and IL-6) rather than leading to an excessive and improper inflammatory cascade triggered by the over-activation of IL-12, and it is a key feature critical for a proper balance of immune activation and tissue homeostasis [12,20,21]. Specifically, the relatively modest IL-12 elevation, when contrasted with robust TNF-α and IL-6 induction, implies that RRBP may engage signaling pathways that preferentially activate NF-κB-driven inflammatory responses while limiting IRF-dependent IL-12 transcription. This selective cytokine profile is biologically advantageous, as it suggests that RRBP can potentiate innate immune defense without excessively skewing toward Th1-polarized inflammatory pathology—a desirable feature for functional food applications requiring controlled and balanced immunomodulation. Furthermore, elevated transcription of iNOS as well as cytokine genes (Figure 6A–D) highlights RRBP’s ability to directly modulate immune responses at the genetic level, thus potentially offering a dual mechanism of action that enhances both innate immune defense and regulatory precision. Accordingly, our observation of the immunostimulatory effect of rice bran polysaccharides supports and is consistent with the earlier findings but our findings also demonstrate the potent activity of RRBP in modulating innate immune responses. For example, our findings align with prior reports on the immunostimulatory properties of rice bran polysaccharides but also reveal novel insights. For instance, experimental findings align with prior studies demonstrating cereal β-glucans induce NF-κB nuclear migration and subsequent nitric oxide/TNF-α production [22,23]. However, the attenuated IL-12 activation observed in our study (Figure 5C) contrasts sharply with the robust cytokine cascades induced by linear β-(1→3)-glucans or mannan/xylan-enriched heteroglycans [24]. Our data suggest that RRBP’s unique structural signature (an α-(1→4)-glucan core interspersed with (1→3,4)- and (1→4,6)-branching nodes) may introduce spatial hindrance that limits optimal IL-12 gene activation. In addition, the induction of iNOS with an exponential dose–response (Figure 6A) offered some insight into the observed mechanism and is consistent with the literature showing that β-glucans stimulate NO production via gene expression of iNOS [12,25].

**Figure 7:**
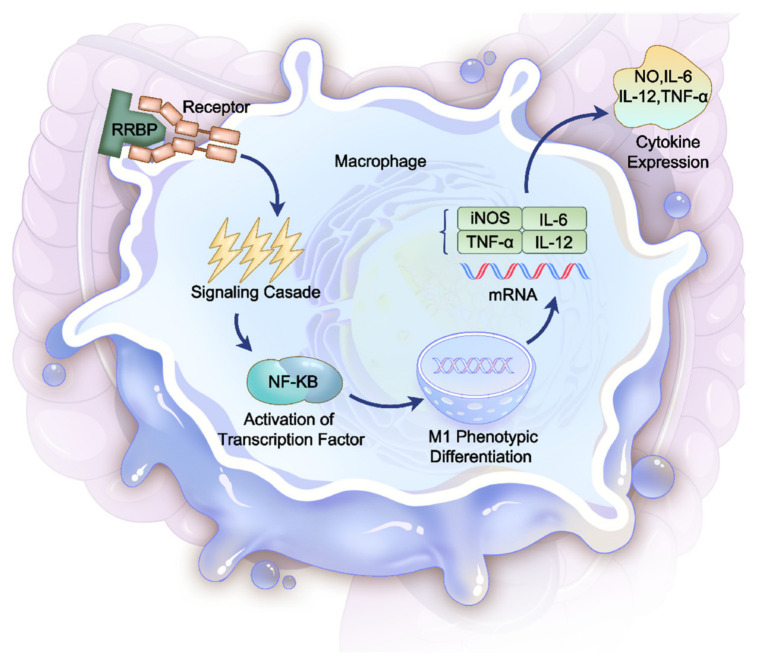
A hypothetical schematic model for the potential mechanism of RRBP-induced macrophage immunomodulation.

Figure 7 illustrates the proposed molecular mechanism by which RRBP elicited macrophage immunomodulation. Firstly, RRBP interacts with a currently unidentified pattern-recognition receptor and initiates divergent signaling pathways such as NF-κB. Then, the gene expression of inflammatory mediators was induced at the transcript level; therefore, the pro-inflammatory mediators NO, IL-6, IL-12 and TNF-α were secreted. This hypothetical model suggests that persistent activation of NF-κB-mediated transcriptional regulation may occur, potentially accounting for the observed upregulation of pro-inflammatory mediators, while simultaneously exhibiting specific suppression of IL-12 amplification pathways. This dual regulatory process culminates in the development of macrophage activation characteristics that mirror M1 polarization features while maintaining intrinsic regulatory constraints, thereby establishing an auto-regulatory inflammatory response system.

Nevertheless, this study elucidates how RRBP regulates the immune system despite those limitations of methodology. Firstly, the application of an immortalized mouse macrophage cell line, in spite of being easy to study at a larger scale, does not account for the fact that in living organisms, different immune cells interact with each other such as CD4^+^ T cells, to form the cytokine network [26].

Nevertheless, while this investigation clarifies RRBP’s immune-regulatory pathways, certain methodological constraints require acknowledgment. Foremost, the predominant use of an immortalized mouse macrophage cell line, although practical for large-scale experimentation, overlooks the complex interactions between varied immune cell populations (including CD4^+^ T-helper cells, regulatory T cells, and natural-killer cells) that collectively shape cytokine networks in living organisms [26]. In addition, future integrated structural studies using high-resolution such as NMR spectroscopy, glycan microarray binding assays and saturation-transfer difference (STD)–NMR for quantification of specific binding will be needed to pinpoint molecular interactions that will have the ability to uncouple NF-κB and IRF signaling pathways [12,18].

## 4. Conclusions

In this study, a novel water-soluble polysaccharide (RRBP) was extracted and purified from red rice bran, and its structural features and immunomodulatory properties were systematically characterized. RRBP is a hyperbranched α-glucan with a molecular weight of 51.4 kDa, consisting predominantly of glucose (98.1%) with a →4)-α-D-Glcp-(1→ backbone (30.6%) and 11 distinct glycosidic linkage types, including multiple branching nodes (3,4-, 4,6-, and 2,4,6-linked Glcp) that contribute to a branching density of 16.63%. Functional assays demonstrated that RRBP promotes M1 macrophage polarization in a concentration-dependent manner without exhibiting cytotoxicity, and stimulates the production of NO, TNF-α, and IL-6 while eliciting only minimal IL-12 secretion. This selective cytokine profile, together with the upregulation of iNOS and pro-inflammatory cytokine gene expression, indicates that RRBP exerts a controlled and balanced immunostimulatory effect on macrophages. These findings suggest that RRBP is a structurally distinctive glucan with a unique immunomodulatory signature. Future studies are warranted to investigate the upstream signaling pathways underlying RRBP’s effects and to evaluate its activity in appropriate in vivo models.

## Figures and Tables

**Table 1:** Structural linkages and partially methylated sugar derivatives in RRBP.

Linkage Type	Methylated Sugars	Mass Fragments (*m*/*z*)	Molar Ratio (%)
t-Glc(p)	1,5-di-O-acetyl-2,3,4,6-tetra-O-methyl glucitol	87, 102, 118, 129, 145, 161, 162, 205	33.36
3-Glc(p)	1,3,5-tri-O-acetyl-2,4,6-tri-O-methyl glucitol	87, 101, 118, 129, 161, 202, 234	4.83
2-Glc(p)	1,2,5-tri-O-acetyl-3,4,6-tri-O-methyl glucitol	88, 101, 129, 130, 161, 190, 205	2.91
6-Glc(p)	1,5,6-tri-O-acetyl-2,3,4-tri-O-methyl glucitol	87, 99, 102, 118, 129, 162, 189, 233	11.64
4-Glc(p)	1,4,5-tri-O-acetyl-2,3,6-tri-O-methyl glucitol	87, 102, 113, 118, 129, 162, 233	30.64
3,4-Glc(p)	1,3,4,5-tetra-O-acetyl-2,6-di-O-methyl glucitol	87, 118, 129, 143, 185, 203, 305	1.47
2,4-Glc(p)	1,2,4,5-tetra-O-acetyl-3,6-di-O-methyl glucitol	87, 88, 99, 113, 130, 190, 233	2.16
3,6-Glc(p)	1,3,5,6-tetra-O-acetyl-2,4-di-O-methyl glucitol	87, 101, 118, 129, 189, 202, 234	1.87
4,6-Glc(p)	1,4,5,6-tetra-O-acetyl-2,3-di-O-methyl glucitol	85, 102, 118, 127, 159, 162, 201, 261	8.66
2,6-Glc(p)	1,2,5,6-tetra-O-acetyl-3,4-di-O-methyl glucitol	87, 88, 99, 100, 129, 130, 189, 190	1.45
2,4,6-Glc(p)	1,2,4,5,6-penta-O-acetyl-3-O-methyl glucitol	71, 88, 99, 117, 127, 130, 137, 190, 261, 338	1.02

## Data Availability

The original contributions presented in this study are included in the article. Further inquiries can be directed to the corresponding authors.

## References

[1] Chen F., Huang S.Y., Huang G.L. (2021). Preparation, activity, and antioxidant mechanism of rice bran polysaccharide. Food Funct..

[2] Wang L., Huang H.Y., Wei Y.Y., Li X.X., Chen Z.X. (2009). Characterization and anti-tumor activities of sulfated polysaccharide SRBPS2a obtained from defatted rice bran. Int. J. Biol. Macromol..

[3] Wang L., Li X.X., Chen Z.X. (2009). Sulfated modification of the polysaccharides obtained from defatted rice bran and their antitumor activities. Int. J. Biol. Macromol..

[4] Liu Q., Cao X.J., Zhuang X.H., Han W., Guo W.Q., Xiong J., Zhang X.L. (2017). Rice bran polysaccharides and oligosaccharides modified by *Grifola frondosa* fermentation: Antioxidant activities and effects on the production of NO. Food Chem..

[5] Park H.Y., Lee K.W., Choi H.D. (2017). Rice bran constituents: Immunomodulatory and therapeutic activities. Food Funct..

[6] Li X., Ren M.F., Zhang X.X., Wang L. (2022). Insoluble dietary fiber (non-starch polysaccharides) from rice bran attenuates cadmium-induced toxicity in mice by modulating the gut microbiota and alleviating liver and kidney injury. Food Biosci..

[7] Liao W., Luo Z., Liu D., Ning Z., Yang J., Ren J. (2015). Structure characterization of a novel polysaccharide from *Dictyophora indusiata* and its macrophage immunomodulatory activities. J. Agric. Food Chem..

[8] Huang R., Zhang J., Xu X., Sun M., Xu L., Kuang H., Xu C., Guo L. (2024). The multiple benefits of bioactive polysaccharides: From the gut to overall health. Trends Food Sci. Technol..

[9] Zhang Y., Li N., Gong H.X., Zhao C.J., Bao X.R., Liu W., Gao J., Zhang J.L., Yin H.S., Dong Z.Q. (2025). Structural characterization and anti-tumor immunomodulatory effects of polysaccharides from *Astragalus mongholicus* with different cultivation modes. Int. J. Biol. Macromol..

[10] Li J., Yang X., Zheng Z. (2025). Immune regulation mechanism of polysaccharide from *Dendrobium officinale* Kimura et Migo on intestinal lamina propria cells. Food Biosci..

[11] Shi D., Xu X., Wang J., Bu T., Sun P., Yang K., Cai M. (2025). Synergistic anti-inflammatory effects of *Ganoderma lucidum* polysaccharide and ganoderic acid A on LPS-induced RAW264.7 cells by inhibition of TLR4/NF-κB activation. Int. J. Biol. Macromol..

[12] Wu D.T., Meng L.Z., Wang L.Y., Lv G.P., Cheong K.L., Hu D.J., Guan J., Zhao J., Li S.P. (2014). Chain conformation and immunomodulatory activity of a hyperbranched polysaccharide from cordyceps sinensis. Carbohydr. Polym..

[13] Ghoneum M., Matsuura M. (2004). Augmentation of macrophage phagocytosis by modified arabinoxylan rice bran (MGN-3/Biobran). Int. J. Immunopathol. Pharmacol..

[14] Kado H., Yoneta Y., Takeo S., Mitsui M., Watanabe N. (1991). Studies on an enzymatically synthesized antitumor polysaccharide SPR-901. Chem. Pharm. Bull..

[15] Chen B., Xia W., Wang X., Qiao Y., Zhou T. (2026). Structural characterization of a novel glucan from rice bran, evaluation of its potential antioxidant and anti-allergy activities. Food Chem..

[16] Fang H.Y., Chen Y.K., Chen H.H., Lin S.Y., Fang Y.T. (2012). Immunomodulatory effects of feruloylated oligosaccharides from rice bran. Food Chem..

[17] Fan Y.G., Hu C.W., Chu C. (2012). Effect of barley β-glucan on murine RAW264.7 macrophages against virulent *Salmonella enterica* serovar Typhimurium. Food Res. Int..

[18] Synytsya A., Novák M. (2013). Structural diversity of fungal glucans. Carbohydr. Polym..

[19] Gao Z., Liu X., Yu J., Li Z., Shi H., Zhang G., Ling J. (2025). Structural basis of immunomodulation by edible fungal polysaccharides: From molecular characteristics to action mechanisms. Carbohydr. Res..

[20] Samuelsen A.B.C., Schrezenmeir J., Knutsen S.H. (2014). Effects of orally administered yeast-derived beta-glucans: A review. Mol. Nutr. Food Res..

[21] Wu S.J., Lu T.M., Lai M.N., Ng L.T. (2013). Immunomodulatory activities of medicinal mushroom *Grifola frondosa* extract and its bioactive constituent. Am. J. Chin. Med..

[22] Goodridge H.S., Reyes C.N., Becker C.A., Katsumoto T.R., Ma J., Wolf A.J., Bose N., Chan A.S.H., Magee A.S., Danielson M.E. (2009). Activation of the innate immune receptor Dectin-1 upon formation of a ‘phagocytic synapse’. Nature.

[23] Vetvicka V., Vetvickova J. (2014). Immune-enhancing effects of Maitake (*Grifola frondosa*) and Shiitake (*Lentinula edodes*) extracts. Ann. Transl. Med..

[24] Ren Y., Bai Y., Zhang Z., Cai W., Del Rio Flores A. (2019). The Preparation and Structure Analysis Methods of Natural Polysaccharides of Plants and Fungi: A Review of Recent Development. Molecules.

[25] Kim H.M., Han S.B., Oh G.T., Kim Y.H., Yoo I.D. (1996). Stimulation of humoral and cell mediated immunity by polysaccharide from mushroom *Phellinus linteus*. Int. J. Immunopharmacol..

[26] Mantovani A., Biswas S.K., Galdiero M.R., Sica A., Locati M. (2013). Macrophage plasticity and polarization in tissue repair and remodelling. J. Pathol..

